# Fixing the Broken Phosphorus Cycle: Wastewater Remediation by Microalgal Polyphosphates

**DOI:** 10.3389/fpls.2020.00982

**Published:** 2020-06-30

**Authors:** Stephen P. Slocombe, Tatiana Zúñiga-Burgos, Lili Chu, Nicola J. Wood, Miller Alonso Camargo-Valero, Alison Baker

**Affiliations:** ^1^Centre for Plant Sciences and Astbury Centre for Structural Molecular Biology, Faculty of Biological Sciences, School of Molecular and Cellular Biology, University of Leeds, Leeds, United Kingdom; ^2^BioResource Systems Research Group, School of Civil Engineering, University of Leeds, Leeds, United Kingdom; ^3^Centre for Doctoral Training in Bioenergy, School of Chemical and Process Engineering, University of Leeds, Leeds, United Kingdom; ^4^Departamento de Ingeniería Química, Universidad Nacional de Colombia, Manizales, Colombia

**Keywords:** acidocalcisomes, biofuel, biomass, biogas, phosphorus, polyphosphate, wastewater

## Abstract

Phosphorus (P), in the form of phosphate derived from either inorganic (P_i_) or organic (P_o_) forms is an essential macronutrient for all life. P undergoes a biogeochemical cycle within the environment, but anthropogenic redistribution through inefficient agricultural practice and inadequate nutrient recovery at wastewater treatment works have resulted in a sustained transfer of P from rock deposits to land and aquatic environments. Our present and near future supply of P is primarily mined from rock P reserves in a limited number of geographical regions. To help ensure that this resource is adequate for humanity’s food security, an energy-efficient means of recovering P from waste and recycling it for agriculture is required. This will also help to address excess discharge to water bodies and the resulting eutrophication. Microalgae possess the advantage of polymeric inorganic polyphosphate (PolyP) storage which can potentially operate simultaneously with remediation of waste nitrogen and phosphorus streams and flue gases (CO_2_, SO_x_, and NO_x_). Having high productivity in photoautotrophic, mixotrophic or heterotrophic growth modes, they can be harnessed in wastewater remediation strategies for biofuel production either directly (biodiesel) or in conjunction with anaerobic digestion (biogas) or dark fermentation (biohydrogen). Regulation of algal P uptake, storage, and mobilization is intertwined with the cellular status of other macronutrients (e.g., nitrogen and sulphur) in addition to the manufacture of other storage products (e.g., carbohydrate and lipids) or macromolecules (e.g., cell wall). A greater understanding of controlling factors in this complex interaction is required to facilitate and improve P control, recovery, and reuse from waste streams. The best understood algal genetic model is *Chlamydomonas reinhardtii* in terms of utility and shared resources. It also displays mixotrophic growth and advantageously, species of this genus are often found growing in wastewater treatment plants. In this review, we focus primarily on the molecular and genetic aspects of PolyP production or turnover and place this knowledge in the context of wastewater remediation and highlight developments and challenges in this field.

## Introduction

### The Problem

Compared with the other macronutrients (carbon, sulphur, and nitrogen: C, S, and N), the biogeochemical cycle of Phosphorus (P) lacks a gaseous atmospheric component to assist with cyclic replenishment of soils (e.g., for N: lightning, biological N-fixation, and the Haber-Bosch process). This has led to the P cycle being described as “broken,” consequently modern agriculture largely depends on non-renewable inorganic Phosphate (P_i_)-based fertilizers derived from geological sources (Elser and Bennett, 2011). High grade P_i_ rock reserves are projected to last for only 50 to 150 years as mined products already show diminished P_i_ content and greater levels of heavy metal contamination. Additionally, global distribution of rock phosphate is uneven with most reserves present in just a handful of countries; Morocco and Western Sahara alone hold over 70% of total global reserves (Van Kauwenberg, 2010; U.S. Geological Survey, 2017). As such, much of the world is vulnerable to volatile prices and supply insecurity. An estimated 80% of extracted P_i_ is being lost due to runoff, which in addition to P_i_ present in sewage discharges is detrimental for the environment (Schröder et al., 2011). In addition, P (and other nutrients) are not being effectively recycled back to agriculture from wastes under the current system. Although livestock manure is often returned to soil, this can be polluting whereas the spreading of sewage and/or sewage sludge is restricted by regulations (Schröder et al., 2011). Current wastewater treatment methods are also affected by regulatory concerns and economic cost, therefore alternative approaches for P-remediation that recycle back to agriculture are needed (Baker et al., 2015). In Europe for instance, an analysis of P stores and flows showed that a higher demand for P fertilizer was mainly driven by poor P efficiency (i.e., useful P output as a function of total system P input: 38% overall). Here, regional P imbalance (P surplus) and system P losses were well correlated to total system P inputs (P_i_ fertilizer, manure, sewage sludge, digestate, etc.) and livestock densities, causing unnecessary P accumulation in soils and rivers (Withers et al., 2020).

### A Potential Solution

Microalgae have long been studied for sustainable wastewater treatment (Baker et al., 2015; Li et al., 2019b) and have the added advantage of accumulating polyphosphate granules (PolyP). This means that they could be used to return P_i_ back to soil in slow-release form and help to close the P-cycle (Siebers et al., 2019); although this approach has been indirectly adopted by reusing alga-rich effluents from wastewater treatment systems in crop irrigation (i.e., reuse of effluents from wastewater pond systems), the actual mechanisms controlling algal P uptake from wastewaters are not well understood. In recent years, the genetic model *Chlamydomonas reinhardtii* has become the foremost species for understanding the molecular biology underpinning P-metabolism and the production of PolyP in microalgae. Although other micro-algal species have been better investigated for wastewater treatment (Li et al., 2019b), *Chlamydomonas* sp. are often identified in sewage treatment works (STW) worldwide (see *Microalgal Research Models*). Therefore, the *Chlamydomonas* genus provides a good option for facilitating research and development, from the laboratory to engineered wastewater treatment systems.

In this review we first evaluate the remediation potential of microalgae in closing the P-cycle, focusing on wastewater treatment. Second, we explore PolyP in relation to its structure, P-starvation responses, synthesis, and turnover in microalgae. We focus principally on *Chlamydomonas* as a genetic and laboratory model. Our working hypothesis is that better understanding of the cellular regulatory factors governing PolyP metabolism would enable the development of species and strains that are more efficient at P recovery from wastewater, as well as shedding light on key environmental variables controlling luxury P uptake by microalgae that can inform engineering design.

## Microalgal Polyphosphates and Wastewater Remediation

In this section, we examine algal PolyP and how their production might be integrated into wastewater remediation and the return of recycled-P_i_ to soils and crops.

### Microalgal Biomass and Wastewater Remediation

Small-scale microalgal biomass cultivation has been traditionally used for human consumption, particularly *Spirulina* in highly alkaline lakes of Africa (Habib et al., 2008; Slocombe and Benemann, 2016). Today, this cyanobacterium, along with *Chlorella*, comprises the bulk of algae biomass harvested (~30,000 tons dry weight per annum) mostly for dietary supplements (Slocombe and Benemann, 2016). With regards to wastewater remediation, microalgae have been extensively used for the treatment of domestic wastewater worldwide for over a century, particularly in combination with naturally occurring bacteria in wastewater treatment pond systems (Tarayre et al., 2016). Wastewater treatment ponds are very effective at removing organic matter, pathogens, and nutrients (N and P), but overall performance is seasonal and climate dependent. From the late 1950s, large-scale, high-rate algal ponds (HRAP) were developed in California to improve conventional pond performance by optimizing algal biomass growth and predominance over bacteria; nowadays, there are many HRAP systems currently in operation elsewhere, in both tropical and temperate climate countries (Park et al., 2011). To avoid C limitation and elevation of pH, CO_2_ provision is desirable, and this can come from bacterial activity, CO_2_ solubilization from the atmosphere by induced mixing or more effectively, from the injection of flue gases from power plants and industry, or even from combined heat and power (CHP) units burning anaerobically generated biogas at STWs (Acién et al., 2016). Flue gas heat recovery systems can also supply energy to increase reactor temperature as low-grade heat from CHP units, usually at around 40°C, which could be used to extend the growth season and raise productivity for micro-algae at cooler temperate latitudes, but this has not been fully explored.

During the 1970s and 1980s, the focus of research into large-scale algal cultivation shifted away from sewage remediation towards the generation of biofuels (Borowitzka, 2013a). Ironically, waste-water inputs into algal biomass cultivation are now considered necessary to render algal biofuel production economic, therefore a combination of the two processes is seen as a way forward (Park et al., 2011). Downstream processes, including harvesting, dewatering, algal lipid extraction, and esterification, are still bottlenecks contributing much to the total biodiesel production cost (Mata et al., 2010), which leaves anaerobic (co-) digestion of sewage sludge (and algal biomass) as the current most feasible option for biofuel (biogas) production at STWs; however, the resulting nutrient rich digestate liquor (concentrate) is still an economic burden despite its potential for nutrient recycling in agriculture. Further economic benefits arising from co-production of high-value algal side-products, such as the keto-carotenoids or pro-vitamin A are feasible (Borowitzka, 2013b), but restrictions in the use of wastewater as a nutrient source for pharmaceutical or nutraceutical production lines is a major hurdle.

### Wastewater Treatment and Phosphate Recycling

The historical provision of sanitation and sewerage was driven by the need to reduce transmission of waterborne diseases (Public Health protection). Off-site/centralized wastewater treatment and disposal later emerged as an effective strategy to control the presence of nutrients (C, N, and P) in surface waters (Environmental protection) (Lofrano and Brown, 2010). On average, high-income countries treat about 70% of their domestic and industrial wastewater, whereas this drops to 38%, 28%, and 8% in upper middle-income, lower middle-income, and low-income countries, respectively. Globally, over 80% of all wastewater and fecal sludge is disposed of without treatment (WWAP, 2017). Large centralized STWs have introduced on site energy generation *via* anaerobic digestion of sewage sludge as a measure to reduce net greenhouse gas (GHG) emissions and energy bills, with Western Europe ahead of this trend in industrialized regions, and India and China as leaders in developing countries (Abbasi et al., 2012). Comparatively little progress has been made to achieve similar efficiencies in the N and P cycles at STWs however, where nutrient control is prioritized over recovery and reuse.

**Figure 1:**
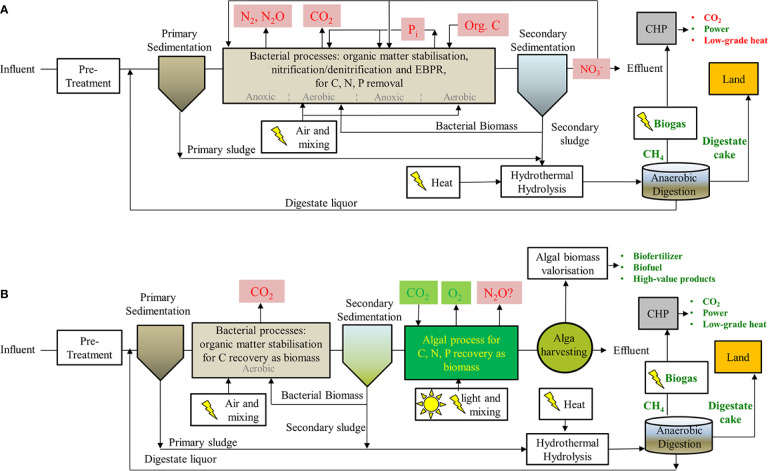
Diagram showing wastewater processing steps for **(A)** conventional STW combining denitrification and EPBR treatments downstream of primary sedimentation and **(B)** proposed substitution of these steps with a microalgae/bacteria consortium. Undesirable products such as important greenhouse gases (e.g., CO_2_, N_2_O), energetically wasteful steps and costly/non-renewable inputs (e.g., supplements: organic C) or wasteful outputs (e.g., N_2,_ NO_3_^−^) are shown in red. Products or steps that are beneficial or that can be potentially renewable are shown in green along with biogas, biofuels, and value products. Downstream anaerobic digestions steps are indicated for both processes resulting in the production of methane and fertilizers.

In the UK, nutrient losses to surface waters are estimated at 980 kt N year^−1^ of total dissolved N and 16 kt P year^−1^ of total phosphorus, with a 60% contribution from urban areas despite the introduction of the EU Urban Waste Water Directive in 1991 (Worrall et al., 2009; Worrall et al., 2016). Current STWs efficiently remove organic matter using heterotrophic bacteria but rely on nitrification/denitrification to remove N compounds, a process that is energy intensive and wastefully returns N_2_ (and greenhouses gases, e.g., N_2_O) to the atmosphere. In fact, the estimated N_2_O emission rate at STWs is 4.9 tonnes of CO_2_-e per tonne of N removed (Johnson and Schroepfer, 1964; Camargo Valero et al., 2010a; NGER, 2016). Some of the C and N is also taken up by bacteria and is removed as harvested biomass at secondary sedimentation units (**Figure 1A**).

On the other hand, P control at small STWs is often achieved by chemical precipitation and adsorption, but these processes usually impede reuse (Tarayre et al., 2016). At large STWs, the bacterial route is usually preferred *via* Enhanced Biological Phosphorus Removal (EBPR). This was first implemented in the 1970s where a group of heterotrophic bacteria, called PolyP-accumulating organisms (PAO) are enriched within the activated sludge process with extended aeration. P_i_ removal by PAO bacteria is achieved *via* aerobic, in-cell accumulation of large quantities of Poly-P (Melia et al., 2017) but this process is limited by organic C, so an expensive acetate supplement is often added as part of the treatment (Manyumba et al., 2009).

The C, N, and P taken up by bacteria are removed from wastewaters as surplus activated sludge and sent to anaerobic digesters (AD) for biogas production, where the C uptake is valorized as biomethane and the N and P are released either as reactive inorganic species (ammonium and P_i_) into the aqueous phase (digestate liquor that returns to the head of the treatment works) or as biomass in a solid phase (digestate cake that is preferably spread on land). Complementary processes recovering both N and P from digestate liquors as struvite precipitates (NH_4_MgPO_4_•6H_2_O) have been implemented at industrial scale but are limited by the costs associated with Mg salts and alkali needed to optimize the precipitation (Campos et al., 2019; Gonzales-Morales et al., 2019).

In contrast, sustainable removal of all inorganic and organic N along with P could be achieved using photosynthetic organisms such as microalgae. Microalgae have been identified as a viable option for meeting energy and nutrient recovery goals in STWs, as they comfortably grow in sewage under either heterotrophic or phototrophic conditions, without the need for supplemental nutrient sources, and the resulting algal biomass can be used to enhance biogas production in existing AD digesters or applied directly as a slow-release fertilizer (Usher et al., 2014; Schreiber et al., 2018).

Research conducted in wastewater treatment ponds reports that *Chlamydomonas, Chlorella, Euglena*, and *Scenedesmus* spp. are the most commonly occurring microalgal species in tropical, subtropical, and temperate climates (Abis and Mara, 2005; Ahmadi et al., 2005; Crimp et al., 2018; Florentino et al., 2019), with effective capacity to meet ammonium and P removal targets. Nutrient control and recovery from sewage is achieved *via* algal uptake, which offers a significant benefit over bacteria in that algae accumulate both N and P. Under some environmental/operational conditions the microalgae perform “luxury P uptake,” defined as the uptake of P beyond that required for growth and storage of phosphate within the biomass as PolyP (>1% P dry weight) (Powell et al., 2008). Also, algae preserve their poly PolyP granules for several days, whereas bacteria tend to rapidly re-release their stored P making any scaled-up PolyP storage and processing through to fertilizer far more difficult (Li et al., 2019b).

### Scale-Up of P Uptake in Algal Treatment Plants

Alga-based STWs have proven their ability to effectively recover both N (from ammonium) and P even under outdoor conditions in temperate climate countries (up to 10% N and 3% P in dry algal biomass), as reported in wastewater treatment pond systems in the UK (Camargo-Valero et al., 2010b) and New Zealand (Powell et al., 2011), therefore removing altogether the need for nitrification/de-nitrification and bacterial P removal processes. However, they have a large footprint, and their performance is still seasonal, heavily dependent on weather conditions, and unpredictable (Powell et al., 2008). Full-scale microalgal ponds for waste-water remediation are associated with losses of biomass and remedial productivity, largely down to issues of maintaining effective mixing and the absence of sedimentation units to recover and recycle algal biomass (Sutherland et al., 2020).

Further research is also needed to improve our understanding on luxury P uptake by microalgae and potential negative impacts arising from large scale microalgae cultivation on wastewaters (Usher et al., 2014), including any potential release of harmful greenhouse gases into the atmosphere such as N_2_O (Plouviez et al., 2019). Grazers and algal pathogens are also an issue (McBride et al., 2016), nevertheless, there is evidence that mixtures of different algal species and bacterial in ecosystem consortia can increase robustness against predation and maintain productivity in large-scale HRAP wastewater treatments (Sutherland et al., 2017).

In future, STWs can be developed/retrofitted in places where space is not at a premium. For instance, freshwater algae can be cultivated in floating modules adjacent to coastal cities for sewage treatment (Novoveská et al., 2016). In the meantime, development and implementation of microalgae-based treatment processes at existing large STWs will require additional considerations as they should fit within existing current process layouts. Proposed schemes where access to land is available often involve standard preliminary treatment (screens, grit, sand removal, etc.) and primary clarification. This is followed by anaerobic digestion (e.g., Upflow Anaerobic Sludge Blanket -UASB reactors) and aerobic stabilization, where a microalgae/bacterial consortium benefits from algal oxygen production and nutrient uptake in symbiosis with bacterial stabilization of organic carbon compounds (e.g., HRAP). Next, potential downstream processes for the valorization of algal biomass (biofuel/fertilizer production) and treated wastewater have been suggested in existing literature (Li et al., 2019b). However, the immediate challenge is benefiting from existing assets at large STWs and finding a feasible alternative for intensive algal cultivation (i.e., small footprint and hydraulic retention times in the order of hours), that take advantage of existing well-known activated sludge processes for the stabilization of organic matter and anaerobic digestion of sludge with thermal hydrolysis for enhanced bioenergy production *in situ* (**Figure 1B**).

### Algal Polyphosphates as Slow-Release Fertilizer

PolyPs of biological origin are unbranched linear polymers of inorganic phosphate (P_i_) linked by phospho-anhydride bonds and of variable chain length, ranging from 10s to 100s. These tend to accumulate in vacuoles or acidocalcisomes as granules (Albi and Serrano, 2016). Although higher plants produce organic phosphate (P_o_) in the form of phytate (inositol hexakisphosphate) there is no strong evidence to support the presence of actual PolyP granules in higher plants, despite a recent and extensive search (Zhu et al., 2020). Instead, these can be found in bacteria, fungi, and lower plants, such as algae and the mosses (Seufferheld and Curzi, 2010).

Given the polluting nature of organic manure (Schröder et al., 2011) it is notable that its P-composition consists largely of orthophosphate (P_i_). Particularly piggery manure (Liang et al., 2017) and to a lesser extent dairy (He and Dou, 2010), where phytate (10% of total P_i_) is also present along with pyrophosphate (6%) and PolyP (3%). The expectation is that algal biomass containing remediated P_i_ in the form of PolyP can be returned to soil in a form that can be taken up by crops with minimal wastage. Hence, PolyP in the form of triphosphate (TPP) has been shown to act as a slow-release P-fertilizer, a desirable characteristic that favors utilization by crops and delays leaching into agricultural runoff (McBeath et al., 2007). Evidence suggests that P_i_ from algal biomass applied to soil can be taken up by plants (Siebers et al., 2019). Furthermore, algal biomass is practical for return-to-soil, not requiring tilling or exhibiting N-volatilization or fugitive methane emissions like manures, sludge or digestate (Mulbry et al., 2005).

Manufacturing a purified form of slow-release P fertilizer from algal biomass has not yet been explored. Laboratory extraction methods of PolyP involve a straightforward aqueous boiling step (Martin and Van Mooy, 2013), therefore it might be possible to incorporate this step into procedures for isolating other algal products such as lipids or carotenoids at a cost-effective commercial level. There is evidence that algal PolyP can be taken up in mammalian gut cell lines, so there could be the option of using biomass for bespoke feeds (Gao et al., 2018).

### Microalgal Research Models

As noted, multiple algal species have been found in STWs, including species known for high biomass productivity and species diversity could be a prerequisite for success. *Chlamydomonas reinhardtii* is the best studied species to date however, in terms of photosynthesis, genetics, and molecular biology (Salomé and Merchant, 2019). Heterologous protein expression has been successful in this alga (although there are others), which shows effective protein folding in the plastid (Gong et al., 2011; Tran et al., 2013) as well as protein secretion (Molino et al., 2018). Therefore, there is much potential for biotechnology and high-value side-products in tandem with wastewater remediation.

A practical advantage of *Chlamydomonas reinhardtii* (and other *Chlamydomonas* sp.) is its rapid propagation under mixotrophic conditions, using acetate. For instance, colonies on agar plates can grow within days facilitating the genetics. Growth rate in mixotrophic mode generally exceeds the sum of the heterotrophic and autotrophic rates (Laliberté and de la Noüe, 1993; Smith et al., 2015). Hence, mixotrophic capability can also boost growth on wastewater, e.g., *C. debaryana* shows potential for wastewater treatment in temperate regions subject to annual fluctuations in light supply (Park et al., 2012). In addition, a *Chlamydomonas* sp. was found growing in palm oil mill effluent, indicating further the utility of this genus (Ding et al., 2016). *Chlamydomonas* sp. are widespread and often found in STWs (Thomas and Burger, 1933; Haughey, 1968; Abo-Shehada and Sallal, 1996; Crimp et al., 2018) whereas the natural occurrence of *C. reinhardtii* is limited to a few lacustrine environments in NE US (Pröschold et al., 2005). Whether or not this species can be used for wastewater treatment is debatable, although mutant strains can be found that demonstrate improved potential for remediation (Zhou et al., 2015). It should be noted that *C. reinhardtii* is unlikely to be mated to other species of the genus (Pröschold et al., 2005) but related species would be relatively easy to replicate mutations in.

Although the genetic model *C. reinhardtii*, has not been considered as a natural biofuel species due to its preference for producing carbohydrate, genetic alterations that reduce flux to carbohydrates in favor of oil such as the *sta6* mutation have been found (Blaby et al., 2013; Goodenough et al., 2014). In addition, certain conditions have been shown to produce “liporotunds” which are large cells containing many lipid bodies (Goodson et al., 2011; Ngan et al., 2015). The appearance of these cells appears to be dependent on acetate provision. This is thought to alter the expression of specific pathways (e.g., increasing the glyoxylate cycle) that favor flux to oil production.

## Microalgal Polyphosphates

In this section, we summarize the microalgal PolyP literature in relation to structure, cell biology, P-starvation responses, synthesis, and turnover. Understanding these factors better would enable the development of species and strains that are more efficient at P recovery from wastewater.

### PolyP Structure and Function

PolyP polymers in pure form exist in various physical states such as sols, gels or glass, although it is not known to what extent these dictate the final structure of PolyP of biological origin. For instance, PolyP adopts a very ordered structure in granules found in the vacuoles of yeast and some green algal species (Shebanova et al., 2017). Interactions of PolyP with metal cations such as Ca^2+^ and Mg^2+^ allow the chains to adopt conformations dependent on their metal ion-coordination preferences (Reusch, 2000). Complexes of PolyP with actin and the cell wall have been noted, and recently, proposed for yeast and microalgae in the case of polyhydroxybutyrate (PHB) (Solovchenko et al., 2019). PolyP interacts with PHB to form channels in bacteria for instance (Reusch, 2000) but we could find no evidence for gene homologs of the bacterial PHB synthase in any eukaryotic genome. The presence of a nitrogenous component in purified *Chlorella* PolyP granules was reported in the 1960s (Correll, 1966) but has never been confirmed. In yeast, arginine accumulates alongside PolyP in vacuoles with some evidence reported for an association although these reserves can also act independently (Dürr et al., 1979; Westenberg et al., 1989). Recently, separate nitrogenous granules containing guanine have been identified in *Chlamydomonas* acidocalcisomes (Moudříková et al., 2017) and distinct granule forms have been visualized in these organelles (Goodenough et al., 2019). Therefore, it is possible that accidental co-purification of separate N-stores might account for some earlier reports of direct PolyP interactions with organic or nitrogenous compounds.

The principal role for PolyP appears to be dynamic P-storage in microalgae. In *Chlamydomonas*, the majority of P_i_ taken up by pre-P-starved cells is incorporated into PolyP. Whereas, during P-starvation PolyP is removed below detection levels along with declines in P_i_, ATP, and sugar phosphates (Hebeler et al., 1992). PolyP appears to be stored in granules in vacuole-like acidocalcisomes in this species (see *Storage in Acidocalcisomes*) but has also been reported in cell walls where it might play a role in cytokinesis (Werner et al., 2007). Increases in P-storage as PolyP are elicited by limitation of other nutrients or with P-resupply following P-starvation—so-called luxury storage (see *Regulation of Phosphate Metabolism in Microalgae*). Other forms of stress that are unrelated to nutrient supply can also have an impact on microalgal PolyP levels and chain lengths, however. For instance, synthesis and mobilization of PolyP appear to be involved in the response to changes in pH and osmoregulation in halotolerant microalgae (Weiss et al., 1991; Leitão et al., 1995). In C*hlamydomonas* species, PolyP mobilization appears to play a role in detoxification and export of Cd and Hg (Nishikawa et al., 2003; Samadani et al., 2008). On the other hand, there is evidence for sequestration of essential and/or toxic metals with PolyP or in acidocalcisomes (see *Sequestration of Heavy Metals by PolyP*).

Other roles for PolyP have been identified in prokaryotes for energy storage, stress response, virulence, motility, quorum sensing and in yeast, antioxidant stress response and protein phosphorylation (Rao et al., 2009; Albi and Serrano, 2016; Docampo, 2020). In future, some of these might be found to operate in microalgae. This can be elucidated by examining mutants defective in genes for PolyP synthesis, turnover, and regulation (see *Storage in Acidocalcisomes*, *Synthesis and Turnover*, and *Regulation of Phosphate Metabolism in Microalgae*).

#### Quantification

**Figure 2:**
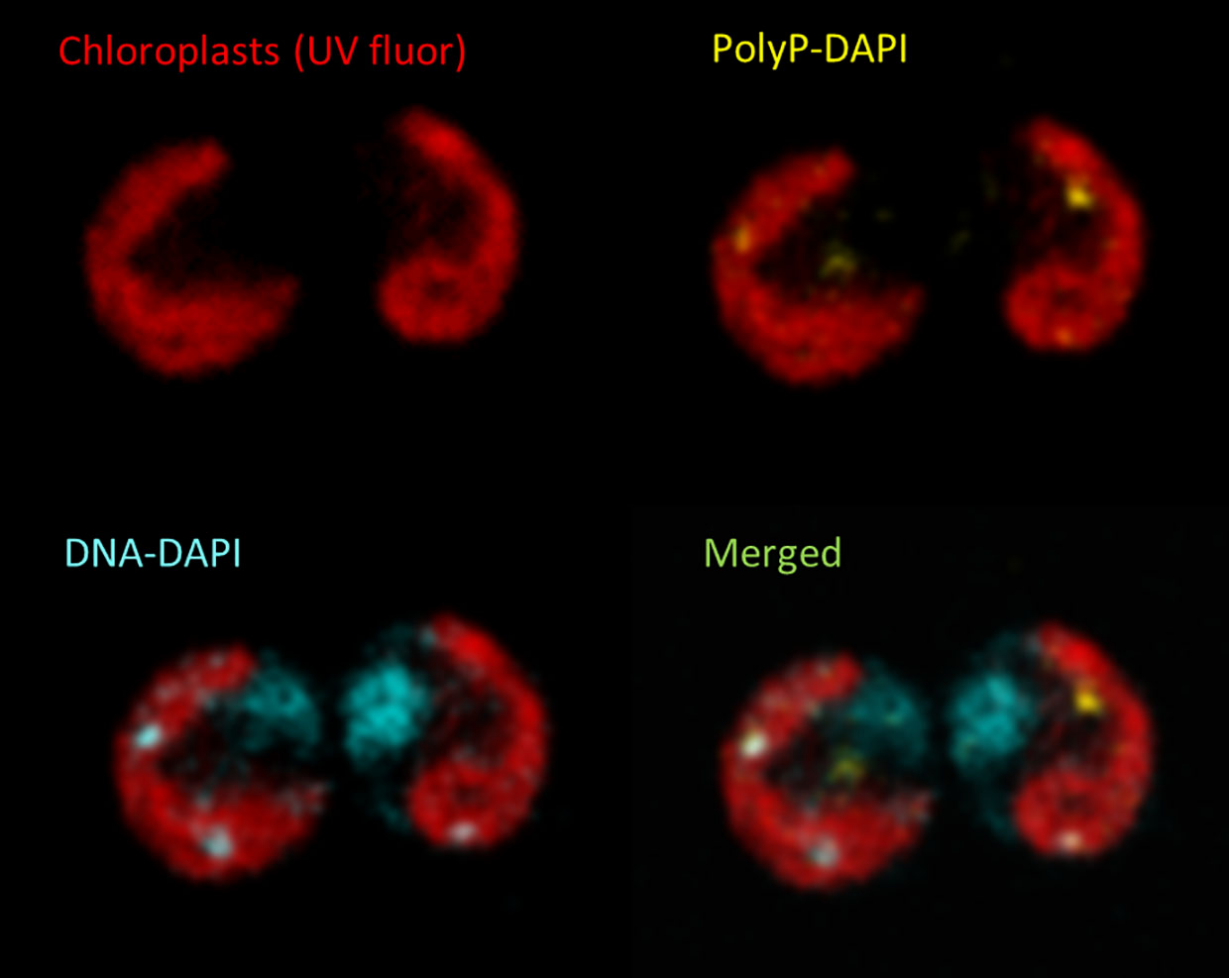
Confocal image of typical live DAPI-stained *Chlamydomonas* cells showing the cup-shaped chloroplast revealed by chlorophyll autofluorescence (red). PolyP granules (yellow) and DNA (blue) were distinguished by the distinct fluorescence of their respective DAPI conjugates. Separate and merged images are shown for the same pair of cells.

Free P_i_ in the microalgal medium is commonly assayed by Molybdate assay (Strickland and Parsons, 1968) and alternatives have been compared (Cogan et al., 1999). Total biomass P can be measured after oxidation and re-mineralization (Solorzano and Sharp, 1980). PolyP granules were originally identified through metachromatic staining (Albi and Serrano, 2016). Today, DAPI staining is often used to visualize PolyP granules in conjunction with confocal microscopy, for example in *Chlamydomonas* (**Figure 2**). Quantification of PolyP was originally achieved by isolating acid-soluble (lower MW) and acid-insoluble fractions (higher MW) using trichloracetic acid (TCA), followed by hydrolysis and assay for P (Aitchison and Butt, 1973). PolyP-DAPI can be used for measurement in plate assays but there is much spectral overlap with DNA-DAPI fluorescence (Shebanova et al., 2017). A PolyP-specific dye has been identified for both microscopy and quantitative plate assays (Angelova et al., 2014) but has only recently been exploited (Zhu et al., 2020)^1^. PolyP can also be separated by chain length by electrophoresis in DNA-sequencing gels or commercial mini-gels (Smith et al., 2018). Enzyme assays can reportedly be used to both quantify PolyP and size chain lengths (Christ et al., 2019).

### Storage in Acidocalcisomes

The principal repositories for PolyP in both eukaryotes and prokaryotes are membrane-bound organelles called acidocalcisomes (Seufferheld et al., 2003; Lander et al., 2016). Five non-exclusive features have been defined for the eukaryotic organelles: i) presence of a single PolyP granule; ii) an acidic lumen; iii) a characteristic complement of trans-membrane transporters (H^+^-PPase and V-type ATPases; metal cation exchangers); iv) a trans-membrane complex responsible for PolyP synthesis (e.g., VTC) and v) a distinctive membrane ultrastructure under TEM which presumably relates to its individual protein/lipid composition (Goodenough et al., 2019). Acidocalcisomes and their PolyP play additional roles beyond storage. For instance, they are required for pathogenicity in eukaryotic parasites, and in *Chlamydomonas* they are central in many stress responses (Docampo et al., 2010). They also have the capacity for Ca-storage (e.g., in *Chlamydomonas* sp., where P_i_ uptake was shown to be Ca-dependent (Siderius et al., 1996)) and this is important for coccolithophore formation in marine Haptophyte algae (Gal et al., 2018). Taken together, functional acidocalcisomes appear to be important for delivering a robust response towards environmental change, with ramifications for outdoor algal cultivation and for micro-organism containment.

In *Chlamydomonas* and Trypanosomes, the organelles are generated at the trans-face of the Golgi (Goodenough et al., 2019). Under N-stress in *Chlamydomonas*, acidocalcisomes generate a single PolyP granule and appear to fuse with autophagous vacuoles (Goodenough et al., 2019). This process could transfer PolyP synthesis capacity to the resultant “hybrid” and account for autophagous vacuoles with multiple granules (Goodenough et al., 2019). It seems the process is also part of a delivery mechanism for periplasmic proteins (Komine et al., 2000; Aksoy et al., 2014) and in Trypanosomes, acidocalcisomes are required for autophagy (Li and He, 2014). PolyP is also likely to be trafficked to the cell wall by this route, where it has a role in retention of some of the periplasmic proteins (Werner et al., 2007). In *Chlamydomonas* the autophagous vacuoles are important in the S deprivation response for macromolecular turnover of S-amino acids and sulfolipids (Aksoy et al., 2014) and seem to be required for TAG synthesis and protein turnover (Couso et al., 2018). A similar process might be occurring in P deprivation where sulfolipids replace phospholipids (see *P-Sparing Measures*). Certain P deprivation–specific periplasmic proteins are delivered by other means however and this route is relatively unimportant during nutrient-repletion (Aksoy et al., 2014). In N deprivation, membrane fragments have been observed in the autophagous vacuoles implying turnover (Goodenough et al., 2019). The autophagy route is dependent on the vacuolar transporter chaperone (VTC) complex which is located at the acidocalcisome membrane and responsible for PolyP synthesis (see *Synthesis and Turnover*) (Aksoy et al., 2014). Together these observations underline the subtle interdependency and partial overlap of responses to the various nutrient-stresses experienced by algae.

#### Sequestration of Heavy Metals by PolyP

Although PolyP/acidocalcisomes are the principal store for Ca^2+^ in *Chlamydomonas* (Siderius et al., 1996) they have been shown to sequester enzyme co-factor cations (Ca^2+^, Mg^2+^, Fe^3+^, Zn^2+^, Mn^2+^) and toxic metals (Al^3+^, Cu^2+^, Cd^2+^ etc.) in a range of organisms (Albi and Serrano, 2016). In *Chlamydomonas*, Cd and Cu toxicity depends on the cellular metal/P ratio, underlining the importance of P-availability in metal-remediation (Wang and Dei, 2006). In this alga, Cu^2+^ co-localizes with PolyP during Zn^2+^ deprivation (Hong-Hermesdorf et al., 2014; Liu et al., 2016) suggesting complex regulation. In some cases, PolyP appear to be necessary for metal uptake in *Chlamydomonas* but are not the final ligand (e.g., for Mn) (Tsednee et al., 2019). This metal-uptake faculty is potentially important for municipal wastewater treatment works receiving industrial inputs that may be contaminated with heavy metals but can affect P recycling for agriculture (Babel and del Mundo Dacera, 2006). This ability might not be universal among microalgae however, since bodies accumulating large amounts of PolyP in another green alga, *Parachlorella kessleri*, were found not to contain the major metals (Ca or Fe) (Ota et al., 2016). Clearly the use of microalgae for both P recovery for agriculture and removal of heavy metals might require separate processes, with some consideration required of the microalgal species.

### Synthesis and Turnover

The pathways for accumulation and mobilization of PolyP in the eukaryotic algal cell are very poorly characterised. *Chlamydomonas reinhardtii* is an attractive model system for addressing this knowledge gap as it is the most intensively studied species. Genetic analysis is facilitated by the existence of 2 mating types, analogous to yeast, and the advanced stages of genome sequence annotation (Salomé and Merchant, 2019). There are extensive collections of point and insertional mutants and gene modification by CRISPR-cas has also been reported (Guzmán-Zapata et al., 2019; Shin et al., 2019) thereby facilitating testing of gene function.

Synthesis of microbial PolyP is carried out principally by reversible ATP-specific kinases PPK1 and to a lesser degree PPK2, which utilizes GTP (Rao et al., 2009). Over-expression of PPK1 in Synechococcus, a cyanobacterium (prokaryotic alga) led to a doubling of PolyP levels (Gao et al., 2018). The occurrence of these genes is very limited in eukaryotes however (Rao et al., 2009) and no orthologues of these genes could be detected in the *Chlamydomonas* genome. Over-expression of *E.coli* Ppk1 in yeast (Gerasimaite et al., 2014) and Arabidopsis (Zhu et al., 2020) has led to toxicity (possibly due to cytosolic PolyP accumulation) and in the latter case, ectopic PolyP granules were identified.

In place of PPK, a VTC complex located at the vacuole or acidocalcisomes appears to be responsible for PolyP synthesis in yeast (Hothorn et al., 2009) and *Chlamydomonas* (Aksoy et al., 2014). In higher plants the existence of the VTC complex is uncertain, and the presence of large stores of PolyP has been questioned (Zhu et al., 2020).

**Figure 3:**
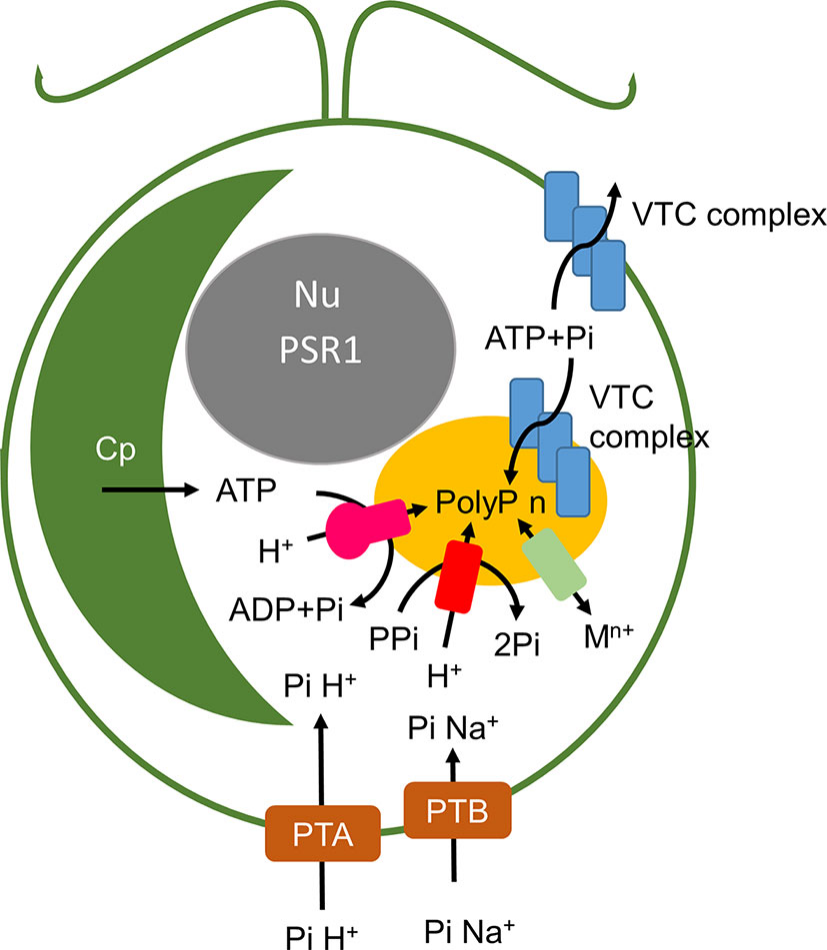
Schematic of polyphosphate synthesis in *Chlamydomonas*. PTA and PTB phosphate transporters are orthologues of the *S. cerevisiae* Pho81 and Pho 89 proton- and sodium-linked transporters and are under *PSR1* regulation as described in the text and **Figure 6**. These are presumed to take up environmental P_i_ under different nutrient and pH levels. The acidocalcisome (yellow) contains a V-type H+ ATPase (pink) and a pyrophosphatase dependent ATPase (red) which energize the membrane. Polyphosphate is synthesized from ATP and P_i_ by the VTC complex. VTC complex and PolyP have also been reported in the cell wall. Metal transporters (pale green) provide metal ion homeostasis. Cp chloroplast, Nu nucleus.

**Figure 3** shows a schematic of poly P synthesis based on the yeast model (with putative or known *Chlamydomonas* orthologues indicated). Two VTC complexes are evident in yeast where one relocates to the acidocalcisomes under P deprivation (**Figure 3**). Electron microscopy (TEMs) in microalgae suggest that a complex at the membrane of this organelle generates strands of PolyP that are injected into the organelle (Shebanova et al., 2017). In yeast, synthesis and translocation by VTC into the organelle probably ensures that PolyP is targeted exclusively to this organelle, given that it appears to be toxic in the cytosol (Gerasimaite et al., 2014).

The acidity of the acidocalcisome lumen is controlled by the proton-pump activity of the membrane-bound H^+^-PPase and the V-type ATPase, where the acidic lumen facilitates cation import by proton-exchangers (Ruiz et al., 2001; Docampo et al., 2010). Lumen acidity could potentially be regulated by stresses such as starvation leading to alterations in acidocalcisome proton-pump activity or expression levels; possibly influencing PolyP synthesis and turnover, and the autophagy process (Li and He, 2014).

Mobilization of P_i_ from PolyP reserves occurs in response to P-starvation in *Chlamydomonas* species (Hebeler et al., 1992; Nishikawa et al., 2006). The key enzymes responsible for PolyP degradation are exopolyphosphatases (PPX) where the prokaryote and eukaryote PPX classes are unrelated. Endopolyphosphatases (PPN) have also been identified in yeast (Albi and Serrano, 2016). The enzymes involved in the mobilization of PolyP have yet to be fully studied in microalgae, although various polyphosphatases have been identified in higher plants (Zhu et al., 2020). Understanding these factors will help target genes for increasing PolyP levels in microalgae for P-remediation.

## Regulation of Phosphate Metabolism in Microalgae

**Figure 4:**
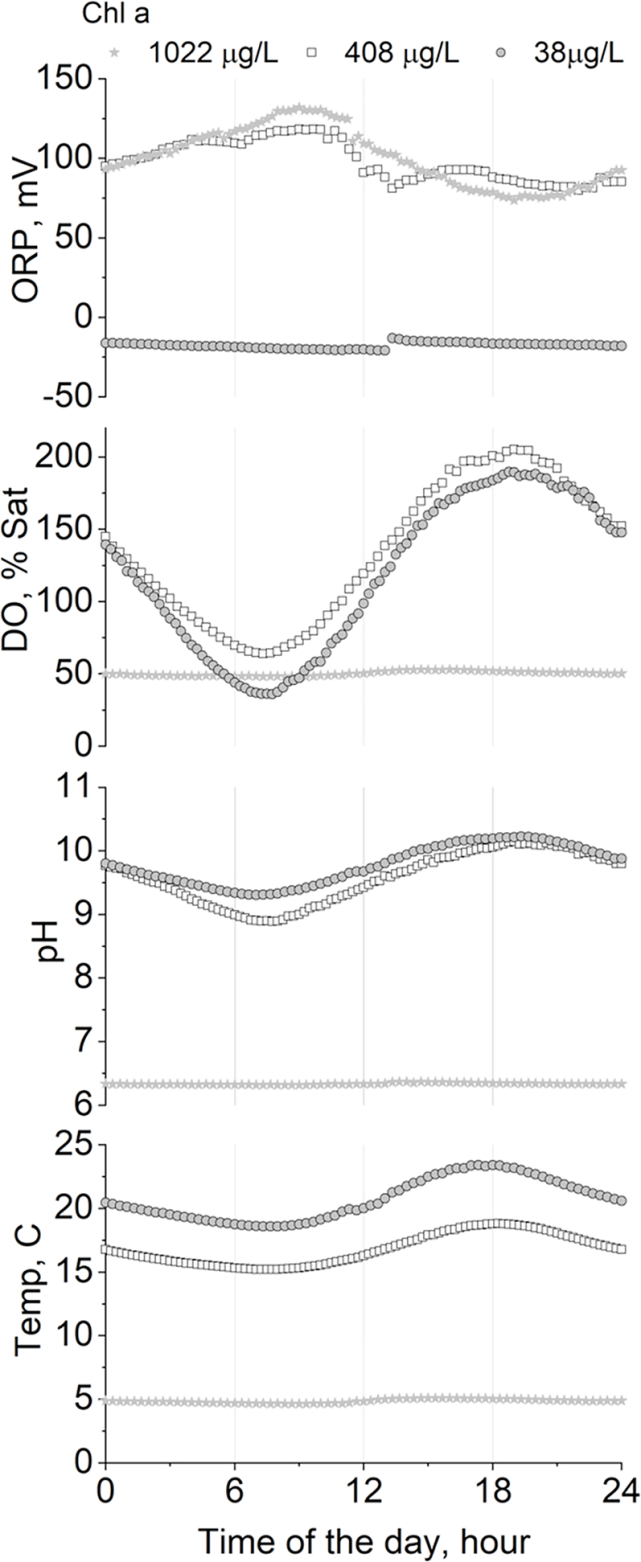
Diurnal variation of pH, oxygen saturation (%), and redox potential (ORP) as a function of algal biomass concentration (reported as Chlorophyll *a* in ug/L) in outdoor algal ponds treating real wastewater in a mix alga-bacteria culture in the UK. Diurnal water temperature variation is also reported for reference (original data from Camargo-Valero, 2009). Published with permission from Miller Alonso Camargo-Valero (co-author) under Creative Commons License (University of Leeds).

In the natural environment, microalgae continually adapt to changing levels of light and nutrients in order to compete for survival, and in doing so change the local environment. For instance, growth on nitrates leads to higher external pH; ammonium to lower pH whereas urea has no effect. Unlike higher plants, algae cannot select N-source to regulate pH but are compelled to utilize ammonium first (Scherholz and Curtis, 2013), which will have the benefit to remove the need for ammonia nitrification at STWs. Microalgae additionally cause diurnal variations in culture pH owing to the uptake and release of CO_2_ dependent on the availability of solar energy for photosynthesis and water temperature, which creates a distinctive variation in dissolved oxygen resulting in direct changes of redox potential (**Figure 4**) and hence, process conditions must be fully understood and monitored at full-scale wastewater treatment systems. Provision of CO_2_ (e.g., from flue gases) or from bicarbonate can regulate culture pH, with the additional aim of removing C-limitation and boosting productivity (Acién et al., 2016). Culture pH can in turn influence P_i_ availability and uptake (Kube et al., 2018). Therefore, the regulation of P_i_ is intertwined with that of other macro- and micro-nutrients.

**Figure 5:**
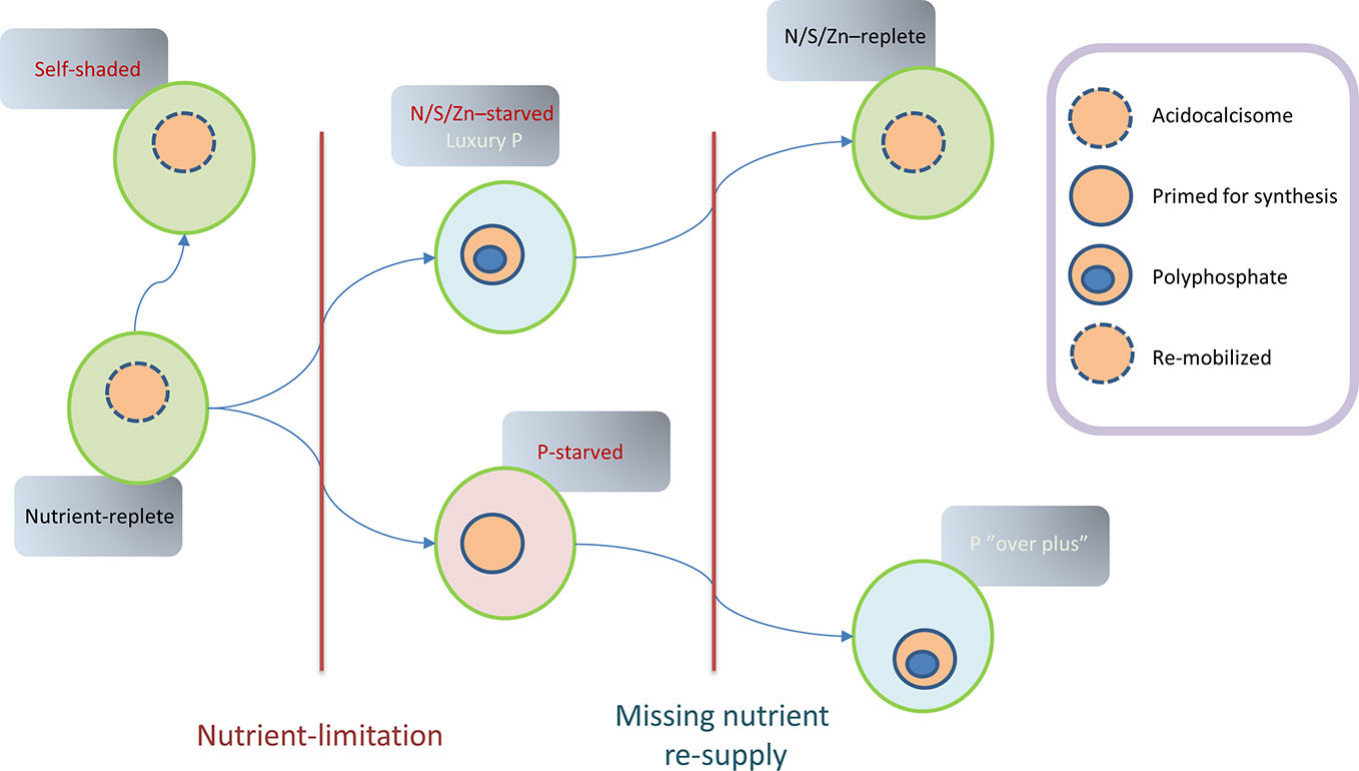
Theoretical model summarizing *Chlamydomonas* P-status during various states that could follow an established period of nutrient-replete growth. In this original state, polyphosphate granules are rare although acidocalcisomes are present. Non-limited growth can lead to self-shading in the presence of abundant nutrients, with nutrient storage perhaps compromised by low light availability. Limited nutrients (N/S/Zn) with replete P lead to luxury P-uptake and PolyP production which can be remobilized if nutrient repletion is restored. In contrast, P-limitation can lead to P-starvation, with measures implemented (e.g., *via PSR1*) to both conserve phosphate and maintain growth leading to a lowering of total P-content. Subsequent P-resupply leads to P “overplus” due to priming for P-accumulation.

The regulation of microalgal P-metabolism itself is particularly complex, possibly because PolyP can be exploited both as a P-reserve and an energy store (**Figure 5**). Early research led to fundamental discoveries and concepts in P storage, particularly in relation to “luxury uptake” which refers to acquisition beyond the immediate cellular requirements (Droop, 1974; Elrifi and Turpin, 1985), with significant ramifications for the nascent fields of ecology and algal biotechnology. Under nutrient-replete conditions (hence light-limited), many microalgae do not accumulate extensive PolyP reserves, in the absence of prior stresses. For instance, in *Chlamydomonas*, PolyP granules are very rarely reported in acidocalcisomes in these circumstances (Goodenough et al., 2019), and in *Chlorella*, measured PolyP levels were low (Aitchison and Butt, 1973). Under active growth, P_i_ must be assimilated in the form of phospholipids and nucleic acids in preparation for cell division (Zachleder et al., 2016). Synthesis of PolyP under replete conditions might thus be expected to compete with growth and cell division, due to the energetics of Pi-uptake and PolyP synthesis (Rao et al., 2009). Diurnal accumulation of PolyP might occur however, particularly where there are diurnal variations in acquisition or availability (Watanabe et al., 1988; Kimura et al., 1999).

As nutrients are consumed, growth often becomes nutrient-limited and ultimately subject to nutrient-stress, particularly in stationary phase (**Figure 5**). Under these circumstances, in *Chlamydomonas* and in other microalgae, such stressors can be drivers of PolyP accumulation as part of the “luxury-phosphate” response. This accumulation is generally observed in stationary phase and is also triggered by experimental depletion of N, S, Zn, amino-acids (in auxotrophic mutants) or alterations in pH in many micro-organisms (Harold, 1966). In the case of *Chlamydomonas*, transfer to N-depleted medium (or S/Zn-deprived) with adequate P supply triggers the formation of PolyP granules which accumulate in acidocalcisomes (Goodenough et al., 2019). This process appears to be analogous to the accumulation of TAG in response to various stresses that slow growth, given that a multiplicity of nutrient-stresses also triggers PolyP synthesis (in the presence of P_i_) (Bajhaiya et al., 2016). In fact, the two processes are likely to compete in some species and in certain circumstances (particularly if enough P_i_ is present).

In the case of P-limitation or starvation (**Figure 5**), depletion leads to a series of regulatory steps to i) conserve P, ii) accelerate uptake, iii) exploit further external P_o_ resources, iv) allow continued growth, and v) prime the cell for P_i_ hyper-accumulation if P_i_ is resupplied (Wykoff et al., 1999). The capacity to produce some PolyP (or turnover) could be important during P deprivation since a knockout of *VTC1* in *Chlamydomonas* was detrimental to growth under these conditions but not under replete conditions (Mahmoud-Aly et al., 2018). The starvation processes appear to be coordinated by a signal-transduction pathway leading to induction of a “Phosphate Starvation Response” myb-type transcription factor *PSR1*. Hyper-accumulation of PolyP or “overplus” was first studied in *Chlorella*, where it was dependent on the duration of starvation before resupply of P_i_ (Aitchison and Butt, 1973). This priming for hyper-accumulation, through induction of PolyP synthesis and P-uptake is presumably a strategic adaptation in the face of fluctuating supplies (Nishikawa et al., 2006; Moseley and Grossman, 2009). This phenomenon is important for P-remediation purposes because it implies that uptake can be accelerated through genetic or physiological means.

### Global Regulatory Networks and *PSR1*

**Figure 6:**
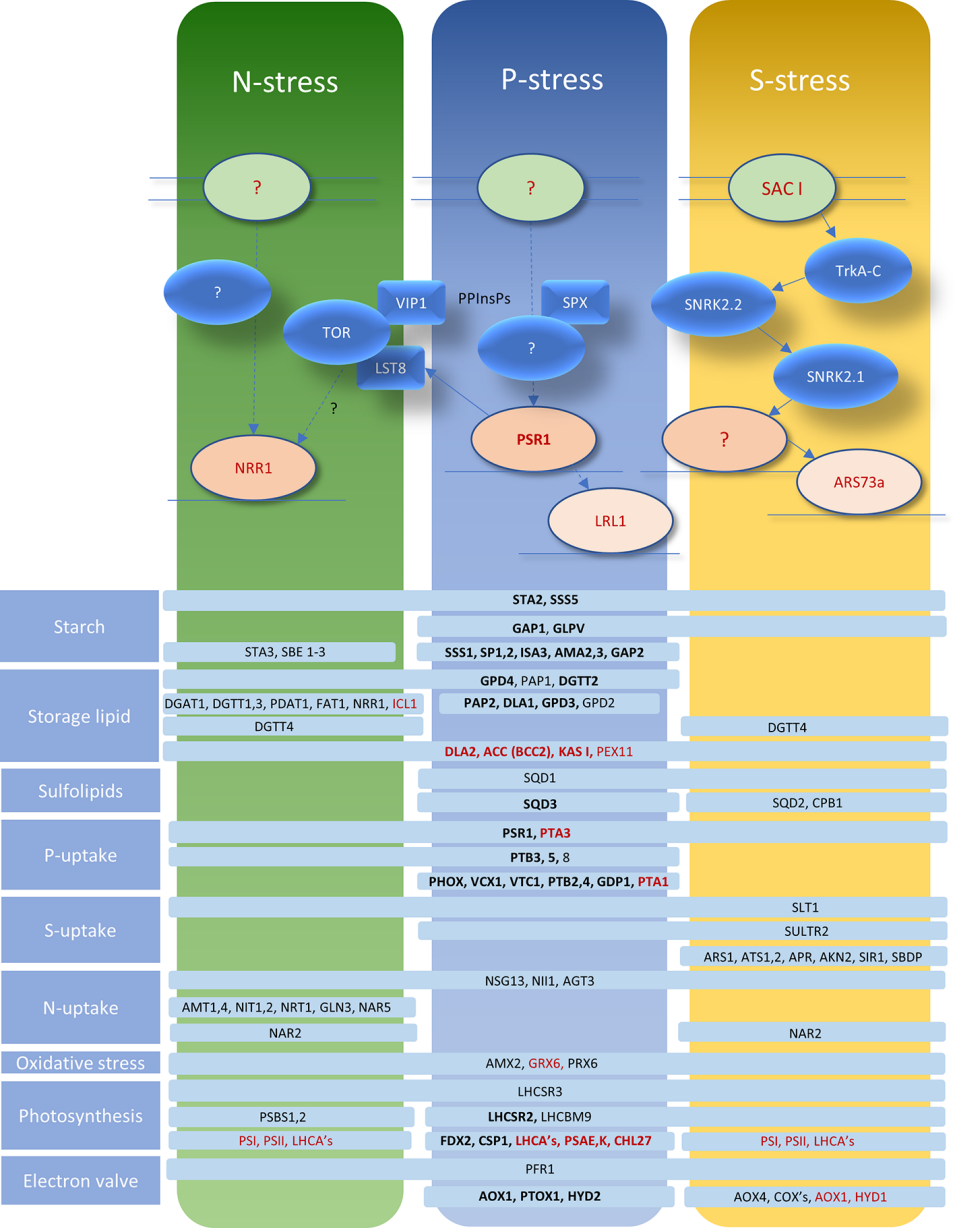
Representation of signal transduction networks for P/S/N deprivation responses in relation to the global regulator *PSR1*. Putative relationships are shown by dotted lines, epistatic relationships by complete arrows. Sensors are indicated by pale green ovals; protein kinases by blue ovals; other signal-transduction factors/domains by blue lozenges, transcription factors are indicated in shades of red and PPInsPs are pyrophosphorylated inositol phosphates. Representative genes showing alterations in expression are indicated in pale blue bars conveying which stress elicits the changes (black: upregulated; red: downregulated; bold: change dependent on *PSR1* for P deprivation). Functional gene categories are indicated to the left. The information and models shown are a collation of transcriptomic data and molecular work from published sources: (Moseley et al., 2006; Gonzalez-Ballester et al., 2008; González-Ballester et al., 2010; Toepel et al., 2011; Schmollinger et al., 2014; Bajhaiya et al., 2016; Wild et al., 2016; Couso et al., 2018; Couso et al., 2020).

The regulatory response pathways for P deprivation are summarized for *Chlamydomonas* in **Figure 6**. These are shown in relation to N and S deprivation, indicating that a degree of overlap exists in terms of gene activation and the putative signal transduction networks. The nature of a sensor that can detect reduced external P_i_ levels is not known but its existence is plausible because the starvation response can be blocked by the P_i_ analog phosphite (Yehudai-Resheff et al., 2007). In yeast and plants, SPX domain proteins (we note that several are encoded in the *Chlamydomonas* genome) bind to pyrophosphorylated inositol phosphates (PPInsPs) which appear to signal intracellular P_i_ levels and influence the P-starvation response and PolyP synthesis (Wild et al., 2016). VIP1 is one such kinase that generates PPInsPs but has been linked to the TOR kinase complex in relation to TAG accumulation and N-deprivation responses (Couso et al., 2018) (**Figure 6**). The LST8 subunit of TOR is down-regulated under P-starvation in a *PSR1*-dependent fashion. This pathway appears to link P-stress to TOR-mediated de-repression of TAG synthesis and autophagy, along with repression of translation (Couso et al., 2020). Investigation of the signal transduction pathway in S deprivation in *Chlamydomonas* has clearly identified SNRK-family involvement (Gonzalez-Ballester et al., 2008; González-Ballester et al., 2010) but this kinase group have also been implicated more widely in other stress responses in this alga (Colina et al., 2019) (**Figure 6**).

The *PSR1* gene was originally found to be instrumental in the “phosphate starvation response” through the phenotypes of its allelic mutants (*psr1-1, 1-2*) which were identified in screens for defects in P_i_ uptake and exophosphatase production (Shimogawara et al., 1999). The mutants showed an inability to divide more than once after imposition of P-stress and a reduction in photosynthesis but were not impeded in their survival of P-stress, suggesting that *PSR1* was responsible for adaptive responses for maintaining growth. When exposed to P-stress, *Chlamydomonas* exhibits a *PSR1*-dependent increase in P_i_-uptake capacity (10-fold increase in the V_max_ with a shift in K_m_ from 10 µM to 0.1–0.3 µM) (Shimogawara et al., 1999). Hence there is an alteration of P importer gene profile (repression of *PTA1,3* and elevation of *PTB 2-5,8*) and increased capacity for PolyP synthesis (*VTC1, VCX1*: encoding respectively a subunit of the transmembrane PolyP synthesis complex and vacuolar Ca-importer). In addition, there is an induction of peri-plasmic phosphatase activity (*PHOX*) to release P_i_ from external P_o_ sources (such as glucose-1-P) (Moseley et al., 2006).

Complementation of this specific phosphatase defect in the *psr1-1* mutant was used to identify the *PSR1* gene as encoding a myb class of transcription factor (Wykoff et al., 1999). Subsequent studies of the phenotypic and transcriptomic responses of the *psr1-1* mutant determined that the *PSR1* gene is required for a wide range of cellular responses to P deprivation and is necessary for many of the gene expression changes associated with these responses (Wykoff et al., 1999; Moseley et al., 2006; Bajhaiya et al., 2016).

#### P-Sparing Measures

Gene expression changes associated with P deprivation include those that directly lead to P-sparing, e.g., turnover and replacement of phospholipids (e.g., phosphodiesterase *GDP1*) with sulfolipids (*SQD1,3*). In addition, there appears to be global *PSR1*-dependent measures hypothesized to reduce P_i_ usage by dialing down the production and consumption of ATP and phosphorylated intermediates (Moseley et al., 2006). For instance, transcriptional repression of genes leading to diminished pentose-phosphate pathway activity along with reductions in chloroplast and cytoplasmic protein translation concomitant with an induction of certain glycolytic steps, suggesting possible regulatory controls (GAP1, 2) (Moseley et al., 2006).

In the chloroplast, DNA levels are reduced in a *PSR1*-independent fashion as a P-conservation measure whereas cpRNA is increased *via PSR1*-mediated repression of a polynucleotide phosphorylase (PNPase) which is phosphorolytic and consumes P_i_ (Yehudai-Resheff et al., 2007).

#### Adaptation to P-Starvation

*PSR1* was also found to be responsible for a range of adaptive responses to lessen the deleterious effects of P deprivation. For instance, retarded cell division and growth due to starvation heralds a greater sensitivity to high light due to photoinhibition through excessive photosynthetic electron energy flux. This is combatted by diminishing photosynthetic function, and photosystem I (PSI) and II abundance. The greatest genetic reductions were seen in Light harvesting complex proteins (LHCs) of PSI (*LHCA*s) and PSI itself (*PSAE, K*), along with chlorophyll synthesis (*CHL27*). Induction of certain LHCs appear to be generally stress-associated (LHCSR2, 3) (Moseley et al., 2006; Greenwell et al., 2010; Ngan et al., 2015; Bajhaiya et al., 2016; Guo et al., 2018). Evidence suggests that the novel LHCSRs dissipate excess light during P-limitation, allowing continued growth (Guo et al., 2018). Many of these changes present an alternative amino-acid composition which might have evolved to enable “sparing” of the elements reduced by stress (e.g., LHCBM9 for S-sparing) (González-Ballester et al., 2010).

Likewise, induction of “electron valve” pathways (*AOX1, PTOX1, HYD2*) provides, respectively, alternative mitochondrial, plastid oxidases, and PSI electron acceptor (Moseley et al., 2006; Bajhaiya et al., 2016), along with alterations in genes associated with oxidative stress (AMX2, PRX6, GRX6) (González-Ballester et al., 2010; Bajhaiya et al., 2016). Diversion of reduced carbon away from growth and into storage reserves also creates a sink for surplus photosynthetic energy. In *Chlamydomonas*, this is mainly achieved through starch production although storage lipids are also induced by P deprivation; where both processes are dependent on *PSR1* (Wykoff et al., 1999; Moseley et al., 2006; Bajhaiya et al., 2016). Here a multiplicity of starch-synthesis genes is induced, principally *STA2* (**Figure 6**) along with a more muted response for the storage-lipid related genes, including a type 2 *DGAT* (*DGTT2*). Upregulation of gene activity is associated primarily with the terminal TAG synthesis steps, rather than the genes for fatty acid biosynthesis, which are either repressed or do not change (**Figure 6**). This is probably an indication that rates of fatty acid synthesis under nutrient-replete conditions are adequate to support the level of TAG synthesis seen with nutrient-stress in this species; either with or without the *sta6* mutation, which prevents starch synthesis and boosts flux to TAG (Blaby et al., 2013). A further MYB TF gene, *LRL1* appears to be influential for maximizing some of the late-response P-deprivation responses (**Figure 6**) (Hidayati et al., 2019).

#### Integration of *PSR1* With Other Networks

Despite the greater research focus on P deprivation, in keeping with the way *PSR1* was initially discovered, its global significance and involvement in other forms of nutrient stresses has been established, suggesting a degree of integration for signal-transduction pathways as a network of “hubs” as proposed in *Chlamydomonas* (Gargouri et al., 2015). For instance, the gene appears to be necessary for storage lipid synthesis associated with S and N deprivation (Ngan et al., 2015) as well as for P deprivation (Bajhaiya et al., 2016). In fact, rapid and transient upregulation of the *PSR1* transcript itself is well-documented for all 3 stresses (Wykoff et al., 1999; Ngan et al., 2015; Bajhaiya et al., 2016). For instance, under P deprivation, *PSR1* peaks at 8 h post-stress, preceding a prolonged induction of exo-phosphatase activity (Wykoff et al., 1999). This is consistent with a likely “classical” negative feedback regulation mechanism, as reviewed (Ueda and Yanagisawa, 2019), for PSR1 but the nature of this has yet to be fully resolved. A comparative transcriptomic study reported that only a small minority of transcriptionally altered genes (96 out of 6930 in total) were affected in all 3 stresses (Schmollinger et al., 2014). Nevertheless, many of the genes falling into this category (**Figure 6**) are major actors in the various biochemical pathways affected by stresses, e.g., *NII1* (Nitrite Reductase), *STA2* (starch synthesis), *LHCSR3, PFR1*, (photosynthetic and “electron valve” adaptation to excess light energy transduction) and the major sulfate transporter gene *SLT1*. Conversely down-regulation of *KASI* and *ACC (BCC2)* (fatty acid biosynthesis) is seen along with *PEX11* (peroxisome biogenesis/beta-oxidation) and *PTA3*, a putative low-affinity/high-rate P-transporter (**Figure 6**).

### Over-Expression of Regulatory Factors

In waste remediation of P_i_, it is desirable to prioritize P_i_-uptake and PolyP production. One option is to hijack or enhance the P-starvation response. The easiest way to achieve this is to target regulatory networks, requiring less gene manipulation (Sang et al., 2005). Alterations to a single transcription factor can effect multiple changes to metabolic or regulatory pathways simultaneously (Bajhaiya et al., 2017; Li et al., 2019a). Since *PSR1* has been identified as global regulator of P deprivation and other nutrient stresses, over-expression of this factor has much potential for wastewater remediation by enhancing a suite of genes that could accelerate uptake of P_i_ and perhaps other nutrients and contaminants (Bajhaiya et al., 2016). For instance, biomass total P levels are increased by 5-fold in *PSR1* over-expressing *Chlamydomonas* lines relative to background control (*cw15*) irrespective of the P_i_-supply conditions (Bajhaiya et al., 2016).

Gene over-expression studies indicated that artificial *PSR1* upregulation elicits an increase in biomass P-content along with a number of P deprivation–specific gene-expression responses under nutrient-replete conditions, such as elevated *PHOX* (exophosphatase) and putative high-affinity P_i_-transporters (PBT2, 4) (Bajhaiya et al., 2016). Although the experiments suggest that *PSR1* upregulation alone might be sufficient to elicit the Pi-starvation response, there was still an additional boost with P-starvation, either in the wild-type backgrounds or in the *psr1* mutant (Bajhaiya et al., 2016). Since over-expressed *PSR1* transcript was also elevated further under P-starvation (despite the use of a constitutive gene promoter) it was arguable that the *PSR1* transcript level was still of key importance, although other factors cannot be ruled out.

In addition to PolyP accumulation, *PSR1* also regulates C-storage products that, in the case of oil, could be useful by-products of wastewater remediation, e.g., for biofuels or other purposes. Accumulation of starch and associated synthesis gene transcripts are greatly induced by PSR1-everexpression, along with increased cell size (Bajhaiya et al., 2016). This particular study was unable to show *PSR1*-directed over-expression of storage lipid in *Chlamydomonas* (backgrounds CC125 or *cw15*: cell-wall deficient) nevertheless, a second over-expression study (Ngan et al., 2015) demonstrated a mean 2-fold increase among multiple transformants (relative to the background, 4a+). Here, a sub-population of transformants was comprised of large-celled “liporotunds.” The appearance of “liporotunds” in *Chlamydomonas* generally appears to require provision of acetate; that is, dependent on mixotrophic/photoheterotrophic growth (commonly provided for in *Chlamydomonas* culture) (Goodenough et al., 2014). The presence of the *sta6* mutation is also recognized as another factor for generating “liporotunds” (Goodson et al., 2011).

It has not been established in any of the over-expression studies whether biomass increase in P_i_ involves increases in PolyP granule production – these are largely absent from wild-type nutrient-replete cells (Goodenough et al., 2019). Accumulation of PolyP under replete conditions would be advantageous for wastewater remediation envisaging a return of biomass to agricultural land in potentially “slow-release” form. Likewise, it is unknown if P_i_-uptake, either from P_i_ or release from P_o_ sources, can be accelerated in wastewater, e.g., given the increased secretion of exophosphatases. Furthermore, it is possible that uptake of other nutrients and trace elements are increased relative to wild-type, given the various gene stress-responses indicated in **Figure 6**. Uncertainty was raised if rates of biomass production would be compromised by *PSR1*-overespression, considering the increases seen in storage products and cell size (Ngan et al., 2015) but this was not noted in a similar study (Bajhaiya et al., 2016).

A further potential advantage of *PSR1*-overexpression, or alteration of any other relevant transcription factor, is to gain a greater influence over nutrient remediation rates when C:N:P ratios of wastewater influent fluctuate. Responses to this ratio are highly complex and species-dependent, having led to much research on which algal strain might be best suited for a certain wastewater stream (Kube et al., 2018). The complexity likely arises from differences in the responses to limitation or starvation of specific nutrients, such as luxury uptake and how their signal-transduction responses might impinge on each other. In addition, C-storage energetics are likely to compete with energy-intensive uptake and storage of N and P. For instance, TAG is a more concentrated and calorific C-reserve than starch therefore, preference for TAG over starch might leave less energy for PolyP accumulation, and this systematic choice varies greatly across the Phyla (Slocombe et al., 2015). Therefore, imposing control over global transcription factors could address a key limitation over the current apparent need to match species to wastewater type (Kube et al., 2018; Li et al., 2019b).

## Conclusions and Outlook

Although global P reserves and the environment are currently under pressure from unsustainable use, much can be gained from improvements in agricultural practice and wastewater management. PolyP-containing microalgal biomass appears to be practical as a slow-release fertilizer, therefore microalgae could be used to recycle P from STWs back to agriculture. This provides an opportunity to improve the energetics, effluent purity, and circular economy of STWs combined with outputs such as P-fertilizer, biofuel, and value products.

Central to this aim is a better understanding of PolyP production and its regulation, particularly in the microalgal research tool *Chlamydomonas reinhardtii*. It is uncertain if this species can be used extensively in STWs but it is likely that other species of the genus can be utilized. Algae from other genera must also be studied, since a species mix appears to protect against biotic and abiotic challenges, particularly in open ponds. There is also a wider need to understand the various cellular and metabolic functions of PolyP given that the field has often been neglected. The regulation of P-metabolism appears to be closely integrated into a network of regulatory “hubs” including other forms of metabolic regulation such as S deprivation. One such factor is the *PSR1* gene which can be manipulated to increase P_i_-uptake and biomass PolyP levels.

Remediation of P from STWs is likely to involve large-scale open ponds, whereas waste-streams for high-value products can be envisaged in closed-circulation systems where novel engineered strains might be permitted. Wastewater inputs have been envisaged as a means of rendering algal biofuels sustainable and economic. For instance, co-localization of sewage treatment plants and flue gas sources (e.g., from power stations or cement works) along with suitable terrain for algal ponds are likely to be increasingly explored. At conventional STWs, existing activated sludge processes can focus on the stabilization of organic matter (no nitrification), while secondary effluents can be used to feed intensive algal-based processes aimed at N and P control and recovery *via* biological algal uptake. The resulting algal biomass can be harvested and valorized as a fertilizer (if remaining pathogen content allows) or as a feedstock for biogas production *via* anaerobic co-digestion with surplus activated sludge, taking advantage of thermal hydrolysis processes already available to increase biomethane production and produce a nutrient-rich, pathogen-free digestate cake for reuse in agriculture.

Overall, improving our understanding on how and why luxury P uptake occurs in microalgae is crucial to fill current knowledge gaps on the regulation of algal phosphate uptake and its effective use in the development of effective P recovery strategies. Removal of nutrients from wastewater to meet regulatory limits is also a major benefit arising from the integration of microalgae into STWs, providing added value, that could help drive economic biofuel and biofertilizer production.

## Author Contributions

SS, TZ-B, LC, NW, MC-V, AB: Literature search, writing, image design, proofing.

## Conflict of Interest

The authors declare that the research was conducted in the absence of any commercial or financial relationships that could be construed as a potential conflict of interest.

## Footnotes

1 Only available by synthesis to order, at the time of writing.
